# A Taxonomic Search Engine: Federating taxonomic databases using web services

**DOI:** 10.1186/1471-2105-6-48

**Published:** 2005-03-09

**Authors:** Roderic DM Page

**Affiliations:** 1 Division of Environmental and Evolutionary Biology, Institute of Biomedical and Life Sciences, Graham Kerr Building, University of Glasgow, Glasgow G12 8QQ, UK

## Abstract

**Background:**

The taxonomic name of an organism is a key link between different databases that store information on that organism. However, in the absence of a single, comprehensive database of organism names, individual databases lack an easy means of checking the correctness of a name. Furthermore, the same organism may have more than one name, and the same name may apply to more than one organism.

**Results:**

The Taxonomic Search Engine (TSE) is a web application written in PHP that queries multiple taxonomic databases (ITIS, Index Fungorum, IPNI, NCBI, and uBIO) and summarises the results in a consistent format. It supports "drill-down" queries to retrieve a specific record. The TSE can optionally suggest alternative spellings the user can try. It also acts as a Life Science Identifier (LSID) authority for the source taxonomic databases, providing globally unique identifiers (and associated metadata) for each name.

**Conclusion:**

The Taxonomic Search Engine is available at  and provides a simple demonstration of the potential of the federated approach to providing access to taxonomic names.

## Background

Biological taxonomy provides the central link between diverse items of information about an organism. Given the scientific name of an organism, a researcher can query a wide range of databases for information on that organism's genome, development, morphology, geographic distribution, behaviour, phylogeny, and conservation status. However, the utility of taxonomic names as keys to accessing information is hampered by several factors, notably the lack of a single authoritative list of all taxonomic names [1,2]. In the absence of such a list, databases that make use of taxonomic names have no ready means of validating those names. Consequently, there is no guarantee that taxonomic names stored in different databases will be mutually consistent.

In the absence of a single database of names, one solution is to use a federated approach [3] where multiple, independent databases are queried. Numerous taxonomic databases exist, although each tends to have limited taxonomic and geographic scope, and the degree of interoperability among these databases varies greatly. The NIH/NIAID/Wellcome Trust Workshop on Model Organism Databases [4] defines the minimum level of interoperability as providing a FTP dump of the database contents. The only taxonomic databases currently meeting even this minimum level are the Integrated Taxonomic Information Service (ITIS) [5] and the NCBI Taxonomy [6] databases. Greater degrees of interoperability require the availability of an explicit Application Programming Interface (API) that clients can use to query the database. Each taxonomic database provider has developed their own interface which is typically aimed at a single user with a web browser. Few databases provide an API, or better still, a *documented *API. Taxonomic names themselves have limitations as identifiers in databases [7] due to the existence of multiple names (synonyms) for the same taxon, and the use of the same name to refer to different taxa. For example, the genus *Morus *applies to both an animal (the gannet) and a plant (the mulberry tree). Even species names can be identical – a species of wasp and a species of conifer both share the name *Agathis montana*. Hence, using names alone to link different data sources can be prone to error. As an example, at the time of writing NCBI's LinkOut feature [8] mistakenly links the catfish genus *Loricaria *(tax_id = 52085) to the TreeBASE [9] taxon *Loricaria *(TaxonID = 1305), which is a plant genus (family Compositae).

To avoid ambiguity some form of identifier other than a taxonomic name needs to be employed, such as Digital Object Identifiers (DOIs) [10] or Life Science Identifiers (LSIDs) [11,12]. Given such an identifier a user can unambiguiously refer to a name, and at the same time discover the provenance of that name (i.e., the source database). The use of globally unique identifiers in taxonomy is in its infancy: the use of DOIs has been explored in the context of prokaryote taxonomy [13], but LSIDs have yet to be employed for taxonomic names. Instead most efforts to link taxonomic databases use URLs (e.g., Species 2000 [14]) and NCBI Linkout [8]). However link integration using URLs has serious limitations [15].

Given the lack of a central list of names, and the limitations of names as identifiers, there is a clear need for a taxonomy name service that can validate names and provide unique identifiers [2]. The SPICE project [16,17] has explored the utility of a federated approach to querying taxonomic databases. For each database, SPICE requires that a wrapper is installed on the computer hosting that database. This wrapper communicates natively with the local database to perform a standard set of queries. The central query engine then communicates with each instance of the wrapper using a consistent protocol (e.g., CGI). This approach places much of the burden of interoperability on the source database, which must adapt and install the SPICE wrappers.

This paper describes the Taxonomic Search Engine (TSE), which takes federated approach to the problem of searching for taxonomic names. Unlike the SPICE project, the TSE relies solely on the interfaces made available by the data source. A wrapper is created for each source database, but this resides on the same machine as the TSE. In this way, no special demands are made of the source database. The TSE searches multiple databases for a name, and returns the result in a consistent format. For each name, TSE also creates a LSID, so that each name from each source database has a globally unique identifier.

## Implementation

### Source databases

The TSE uses five data providers: ITIS, Index Fungorum, IPNI, uBIO, and the NCBI.

#### ITIS

The Integrated Taxonomic Information System (ITIS) [5] was established in the mid 1990's by a consortium of United States federal agencies tasked with to providing a database of taxonomic information for North American taxa. In addition to the original site in the United States [5], there is a French language version hosted by the Canadian Biodiversity Information Facility [18], and a Spanish language version hosted in Mexcio [19]. The Canadian site can serve data in XML format, and users can search for a name, or retreive details about an individual record using a simple URL API. A Document Type Definition (DTD) file for the XML format is available from the ITIS web site.

ITIS provides a classification of taxonomic names (i.e., a parent-child hierarchy), and where more than one name exists for a taxon, ITIS specifies which name it regards as correct (termed the "accepted" name if the taxon is an animal, and "valid" if it is a plant). Every name in the database, regardless of taxonomic status or position in the hierarchy is assigned a unique identifier (its "taxon serial number"). The database schema is fully documented, and the entire database is available for downloading by FTP as a SQL schema with the data in delimited text files. As a consequence, ITIS is frequently used as the *de facto *source of taxonomic data in biodiversity informatics projects.

#### IPNI

The International Plant Names Index (IPNI) [20] combines data from three sources: Index Kewensis (Royal Botanic Gardens, Kew), the Gray Card Index (Harvard University Herbaria), and the Australian Plant Names Index (Australian National Herbarium), and contains some 1.6 million records. It provides names and associated basic bibliographical details for vascular plants. The IPNI web site provides web forms for querying the database, and data can be returned in HTML, "%" delimited text, or XML. However, the XML is a serialisation of IPNI database objects, rather than a format designed to be handled by end users. There are plans to support emerging standards, such as the Taxonomic Concept Transfer Schema [21]. IPNI aims to be a catalogue of all names that have been applied to vascular plants. However, where more than one name for a taxon exists, IPNI does not specify which name should be used, that is, it does not indicate an "accepted name" for a taxon. In this sense it is That is, it is a nomenclatural database rather than a taxonomic database. However, if two names are nomenclatural synonyms, the HTML output specifies the nature of synonymy, such as "basionym" (one name is the original name for the taxon), "nomenclatural synonym" (one or other of the names is the basionym, or the names share a basionym), or "replaced synonym" (one name has been created to replace another). IPNI provides a minimal classification, in that genera are assigned to families, but no higher-level classification is given.

#### Index Fungorum

IndexFungorum [22] is a database of over 370,000 names of fungi, primarily at species level. The database can be searched through a web interface or through a SOAP web service  which returns an XML document. If more than one name exists for a fungus, Index Fungorum designates one name as the "current name." It also reports the basionym (first recorded name) for that taxon. Index Fungorum does support a detailed hierarchical classification in the form of a lineage, but higher level taxa are not assigned records in the database (unlike, for example, ITIS). In fungal taxonomy, names are often assigned to the asexual state (anamorph) of a fungus for which the sexual state (telomorph) is unknown. Names for anamorphs are flagged as such in the database.

#### uBio

The Universal Biological Indexer and Organizer (uBio) [23] is a product of the science library community, and is motivated by the information retrieval problem posed by the lack of long term stability of many taxonomic names [2]. Presently it is the single largest electronic catalogue of scientific names (1,396,868 as of 13 November 2004). In addition to a web interface uBio provides a SOAP web service  which returns a nested array data structure.

#### NCBI

The NCBI Taxonomy database [6] is a curated database of the names of all organisms for which sequences have been submitted to GenBank [24]. Each taxon regardless of taxonomic level is assigned a unique identifier (the "taxid"), and the NCBI taxonomy provides a single classification for all taxa in its database. If a taxon has more than one scientific name, each name has name has the same taxid, but only one is indicated as the "scientific name" [25]. The other names are flagged as synonyms, common names, etc. The NCBI taxonomy is not intended to be an authoritative source of taxonomic information, but is a rapidly grouping database that contains many taxa that are not found in other databases. Although every sequence in NCBI is assigned to an organism, in many cases the exact identity of that organism may be unknown. Sequences obtained from environmental sampling are typically unidentified, and the number of such sequences is likely to increase with the advent of large scale environmental genomics [26]. The NCBI taxonomy database can be queried via the Entrez Utilities [27] using wither a URL or a SOAP interface. The entire database is also available for download by FTP.

### Architecture

**Figure 1:**
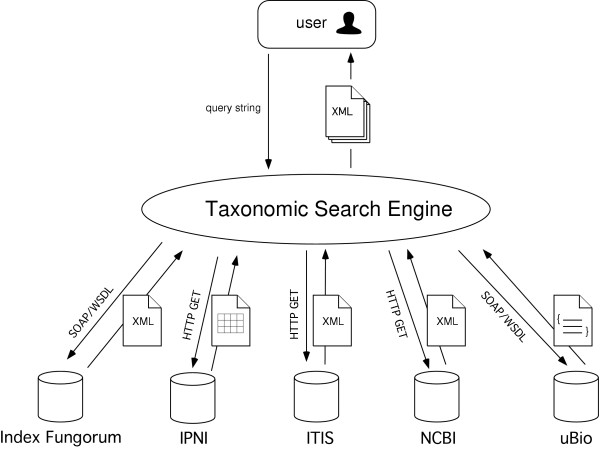
**Architecture of the Taxonomic Search Engine. **The user's query is passed to each database using either the HTTP GET protocol or SOAP, and the results (which may be in XML format, delimited text, or a SOAP data structure) are combined and returned as an XML document.

The basic architecture of the TSE is summarised in Fig. 1. For each database a wrapper (implemented as a class in the PHP scripting language) is responsible for communicating with the database, using either the HTTP GET protocol (using the Net HTTP Client [28] library) or SOAP (using the NuSOAP library [29]). The wrapper takes the query string supplied by the user, and constructs a suitable query for the corresponding database, such as a URL or a SOAP call. The wrapper is also responsible for handling the response. If databases return a XML document this is transformed using an XSLT style sheet into the XML format used by TSE. Other formats such as text or SOAP data structures are converted into XML by the wrapper.

Each wrapper is derived from the same base class which provides some generic routines for creating XML documents and for caching results (see next section). The wrapper class supports three methods, *IsAlive*, *NameSearch*, and *GetDataForID*, which must be overridden in descendant classes. The *IsAlive *method queries whether the data source is available. The *NameSearch *method queries a data source for a given string. If one or more names are found, *NameSearch *returns basic information about that name, including the identifier used by the data source. This identifier is used by the *GetDataForID *method to query the data source for more details about the name.

#### Caching results

In order to improve the responsiveness of the search engine, the results of queries to each source database are cached for 24 hours. The results of the query are stored in the format returned by the database (i.e., XML or delimited text), except for uBio where the SOAP response is serialised to disk.

#### Approximate string matching

The Taxonomic Search Engine seeks exact matches to the user supplied query. In order to accommodate spelling mistakes the web interface to the search engine supports approximate string matching using two techniques. The first employs agrep [30] to search for a match amongst a flat file list of names obtained from the ITIS and NCBI databases. Names showing no more than two character differences from the query string are returned as suggested alternative spellings. To supplement agrep, the TSE calls Google's spelling suggestion web service [31] and adds the result of that query (if any) to the list of suggested spellings.

### Interface

**Figure 2:**
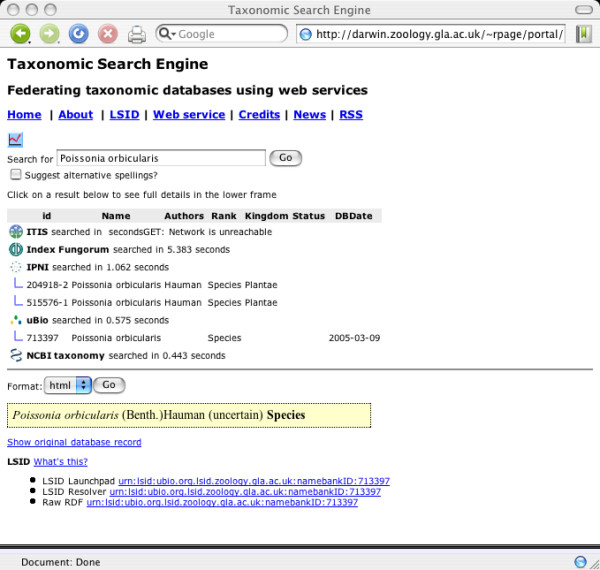
**Screen shot of the Taxonomic Search Engine. **The web browser displays the results of searching for a name in five external databases. For each database that returns a "hit" the page displays some information about that name. The user can click on the name to obtain further information about the name, including a link to the original database record, and a Life Science Identifier (LSID) for that record.

The TSE has a simple web interface (Fig. 2). The user types in a query, and has the option to specify whether TSE should look for alternative spellings. Clicking on the "Go" button starts the search. The XML summary of the search is transformed into HTML using an XSLT transformation. The user can click on a name to get more information, including a link to the original database source for the name, and a LSID for the name.

### Web service

The TSE has a SOAP web service that is described by a Web Services Description Language (WSDL) file available at . The service provides two operations: *NameSearch *which queries the source databases for a user-supplied name, and *SpellingSuggestion*, which suggests alternative spellings for a name. Hence users can write web service clients that can use the TSE as part of their own applications. The TSE web site provides source code for two simple clients written in perl.

### Life Science Identifiers

A LSID is a Uniform Resource Name (URN) comprising five parts: the Network Identifier ("lsid"), the root DNS name of the issuing authority, a namespace, an object identifier, and optionally a revision id to indicate the version [11]. TSE generates LSIDs by concatenating the name of the source web server with the suffix "lsid.zoology.gla.ac.uk" to generate the authority. The namespace is the name given to the identifier in the source database, and the object identifier is the identifier used by the source database. For example, the record for *Homo sapiens *in the ITIS database would have the LSID:

urn:lsid:itis.usda.gov.lsid.zoology.gla.ac.uk:tsn:180092

where "tsn" is the "taxonomic serial number" used by ITIS as a unique identifier for each taxonomic name, and "180092" is the tsn for *Homo sapiens*.

The TSE uses the perl library distributed by IBM's Life Science Identifier project [11] to create a LSID authority for each of the source databases. Hence, any software that can resolve LSIDs (such as LaunchPad [11] or the BioPathways Consortium Web Resolver [32]) can view the metadata associated with an LSID generated by TSE. For ITIS this metadata is constructed by querying a local copy of the ITIS database, but for the remaining databases the LSID metadata is generated using the same combination of GET/HTTP and SOAP calls used to query the source databases by TSE (although these calls are implemented in perl).

### Performance evaluation

The 2004 edition of the Species 2000 CD-ROM [14] was used as a source of names with which to query the TSE. This database comprises 583,469 names provided by 18 taxonomic databases, two of which (ITIS and Index Fungorum) are also source databases for TSE. In addition, uBio currently includes names from the 2003 edition of the Species 2000 CD-ROM in its database. Hence, most names in the Species 2000 list are likely to be found by TSE.

To create a test dataset, 1000 names were selected at random from the Species 2000 dataset. Each name was sent to the TSE web service by a perl script which recorded the time taken for each source database to respond to the query, and whether that source database contained the name. The time recorded is from the time the query was made until the time the response was returned – post processing by the TSE is not included in the measurement. For this experiment, the cache feature was turned off so that for each query the TSE went to the external source database, rather than using a local copy of the query result.

## Results and discussion

### Performance

The results of the simple performance benchmarks are shown in Table 1. Most of the names were found in uBio (887 of the 1000 names), which is as expected given that uBio has harvested all the names in the previous (2003) edition of the Species 2000 CD-ROM. ITIS is a major contributor to both uBio and Species 2000, and just over half the names in the test set are present in ITIS. The Species 2000 CD-ROM contains some names from Index Fungorum, and none from IPNI, hence its coverage of plants and fungi is somewhat limited. That only 10% of the query names were found in the NCBI database suggests there is little overlap between the taxa being catalogued by taxonomic databases and those being sequenced. Amongst the five source databases, ITIS had the slowest median response time (0.915 seconds) and Index Fungorum was the quickest (0.132 seconds). The IPNI database was the second slowest, and occasionally took up to a minute to respond – on 20 occasions no response was obtained at all. It is difficult to generalise about these results as the performance of a data source will depend on a number of factors, such as the server hardware and software, the database design, and the load other users are placing on the system. For the five data sources currently queried, the operating systems being used include both Linux and Windows 2000, the web servers are Apache, Oracle HTTP server, and Microsoft IIS (determined by NetCraft [33]), and the database vendors include Microsoft, Oracle, and MySQL. However, it is encouraging that five such disparate systems all have a median response time of less than a second.

### Extensibility

The TSE can be extended to handle additional data sources simply by deriving a new wrapper class from the base class. To date wrappers have only been written for data sources which can return plain text, XML, or SOAP messages. There are many more taxonomic databases that could be queried if wrappers were written to handle HTML output ("screen scraping"). However, this would make the wrapper very vulnerable to changes in web page design [34]. Of course, a change in a data source's API would also break the wrapper. This is a general problem in integrating disparate databases [34], and in the long term a better solution would be for each taxonomic database to support a standard API that services such as the TSE can query.

### Scalability

Despite the reasonable performance of TSE, there are obvious limitations in the current design and implementation. The PHP language does not support threads, so each source database is queried sequentially. As additional source databases are added the time to complete the search will get progressively longer. If the performance of additional databases is comparable to those already being queried (Table 1), then each new source will add at least 0.5 – 1.0 seconds to the time required for TSE to return a result (not counting the additional overhead of pre- and post-processing the query). If the search engine is to scale to handle a large number of databases it is likely that these databases will need to be queried in parallel.

### Query filtering

Some source databases have broad taxonomic coverage such as ITIS, NCBI, and uBio, whereas others are restricted to particular groups, such as fungi (Index Fungorum) and vascular plants (IPNI). Hence, it makes little sense to query Index Fungorum or IPNI for an animal name (especially as this will could 1–2 seconds onto the time taken to complete the search). An option to select the databases to query could be easily added to the TSE web interface. However, it would be more efficient if the TSE could determine which databases were relevant to the user's query. If the TSE knew that the query string was the name of a fungus, it could send the query to the appropriate database. In practice, however, this is problematic. In order to know what organism a name refers to the TSE would have to have access to a databases of names and their classification – the very lack of such a database is the motivation behind the TSE in the first place. Furthermore, as discussed above, the same name can apply to different organisms. A user searching using the term "Morus" might be looking for a plant name, or an animal name (or perhaps both). There is some scope for more intelligent querying, such as looking for aspects of the name that are specific to one of the codes of nomenclature (e.g., most plant family names end in "-aceae"), but any such effort needs to be done with care – for example, "Compositae" is a family of plants.

## Conclusion

The Taxonomic Search Engine is a simple tool for querying multiple taxonomic databases. Typically, results of querying five major databases are returned in a few seconds. In addition to providing basic information about a name, the TSE acts as a LSID authority, providing globally unique identifiers for each name. The TSE provides a simple demonstration of the potential of the federated approach to providing access to taxonomic names.

## Availability and requirements

The source code for the TSE, the web site, and the LSID authorities is available from the TSE site .

### System requirements

TSE requires a web server and the PHP scripting language. It has been developed and tested under Red Hat Linux 8.0 with the Apache web server version 2.0.40 and PHP version 4.2.2, and Mac OS X 10.2.8 with Apache version 1.3.29 and PHP version 4.3.4. If PHP does not have the XSLT extension enabled then the user will either have to recompile PHP, or install the Sablotron toolkit [35]. The code makes use of various PHP libraries including NuSOAP [29], Net HTTP Client [28], Php.XPath [36], and phpdomxml [37]. The approximate string matching feature requires agrep to be installed (available from ), and a developer key from Google [31].

## Figures and Tables

**Table 1:** Performance of each source database used by the Taxonomic Search Engine. Each database was queried for 1000 taxonomic names taken at random from the 2004 edition of the Species 2000 CD-ROM. The table displays the number of times each database contained the name (*n*), and median, mean, standard deviation, and best and worst times taken for a database to respond to a query. The number of times a query failed to return a response is also recorded.

		Response time (in seconds)					
Source	*n*	Median	Mean	StdDev	Best	Worst	Failed

ITIS	513	0.915	1.151	0.802	0.808	6.593	0
Index Fungorum	73	0.132	0.250	0.562	0.108	9.379	6
IPNI	153	0.356	1.055	3.264	0.143	59.653	20
uBio	887	0.295	0.384	0.544	0.259	8.710	0
NCBI	101	0.252	0.369	0.561	0.225	8.983	0
